# A tale of two multi‐focal therapies for glioblastoma: An antibody targeting ELTD1 and nitrone‐based OKN‐007

**DOI:** 10.1111/jcmm.17133

**Published:** 2021-12-14

**Authors:** Michelle Zalles, Nataliya Smith, Debra Saunders, Megan Lerner, Kar‐Ming Fung, James Battiste, Rheal A. Towner

**Affiliations:** ^1^ Advanced Magnetic Resonance Center Oklahoma Medical Research Foundation Oklahoma City Oklahoma USA; ^2^ Oklahoma Center for Neuroscience University of Oklahoma Health Sciences Center Oklahoma City Oklahoma USA; ^3^ Department of Pathology University of Oklahoma Health Sciences Center Oklahoma City Oklahoma USA; ^4^ SurgeryResearch Laboratory University of Oklahoma Health Sciences Center Oklahoma City Oklahoma USA; ^5^ Stephenson Cancer Center University of Oklahoma Health Sciences Center Oklahoma City Oklahoma USA; ^6^ Department of Neurology University of Oklahoma Health Sciences Center Oklahoma City Oklahoma USA

**Keywords:** angiogenesis, blood perfusion rate (BPR), ELTD1, glioblastoma (GBM), monovalent monoclonal antibody (mmAb), MRI, OKN‐007, orthotopic G55 xenograft model, tumorigenesis

## Abstract

Glioblastoma (GBM) is the most common primary malignant brain tumour in adults. Despite a multimodal treatment response, survival for GBM patients remains between 12 and 15 months. Anti‐ELTD1 antibody therapy is effective in decreasing tumour volumes and increasing animal survival in an orthotopic GBM xenograft. OKN‐007 is a promising chemotherapeutic agent that is effective in various GBM animal models and is currently in two clinical trials. In this study, we sought to compare anti‐ELTD1 and OKN‐007 therapies, as single agents and combined, against bevacizumab, a commonly used therapeutic agent against GBM, in a human G55 xenograft mouse model. MRI was used to monitor tumour growth, and immunohistochemistry (IHC) was used to assess tumour markers for angiogenesis, cell migration and proliferation in the various treatment groups. OKN and anti‐ELTD1 treatments significantly increased animal survival, reduced tumour volumes and normalized the vasculature. Additionally, anti‐ELTD1 was also shown to significantly affect other pro‐angiogenic factors such as Notch1 and VEGFR2. Unlike bevacizumab, anti‐ELTD1 and OKN treatments did not induce a pro‐migratory phenotype within the tumours. Anti‐ELTD1 treatment was shown to be as effective as OKN therapy. Both OKN and anti‐ELTD1 therapies show promise as potential single‐agent multi‐focal therapies for GBM patients.

## 1 INTRODUCTION

Glioblastomas (GBMs) represent approximately 57% of all gliomas and are the most common primary malignant central nervous system (CNS) tumour.^1^ Currently, standard treatment includes surgical resection to remove the bulk tumour, radiotherapy, chemotherapy with temozolomide (TMZ) or bevacizumab, and supportive care.^2^ However, overall survival is poor with a median survival time of 12–15 months.^2^

The current problem lies with the chemotherapeutic agents. Temozolomide (75 mg/m^2^ daily).

is currently given during radiotherapy followed by another 6 cycles of TMZ.^3^ TMZ is an alkylating agent that produces DNA lesions, leading to cell death.^4^ Currently, TMZ is the only approved chemotherapeutic agent that has successfully prolonged the overall survival of patients.^5^ However, resistance to TMZ is a key cause of treatment failure. High expression of O^6^‐methylguanine‐DNA methyl‐transferase (MGMT) induces and contributes to TMZ resistance by restoring tumour cell DNA.

Bevacizumab, a humanized monoclonal antibody therapy against the vascular endothelial growth factor (VEGF), is a biologic that is used to combat GBMs. Bevacizumab selectively binds onto circulating VEGF to inhibit its binding onto a receptor (VEGFR) on the surface of endothelial cells.^6^ Although pre‐clinical studies showed promise, bevacizumab has not significantly increased overall patient survival in newly diagnosed and recurrent GBM patients.^6^, ^7^ Instead, tumours treated with bevacizumab show increased tumour metastasis and invasion alluding to a pro‐migratory phenotype.^8^, ^9^, ^10^ For example, loss of VEGF signalling has led to a more aggressive tumour phenotype in pre‐clinical mouse models.^11^ Clinically, this pro‐migratory phenotype is seen by the development of invasive non‐enhancing tumour progression on MRI.^12^

Tumour angiogenesis is greatly upregulated in human high‐grade gliomas in order to deliver nutrients and oxygen to the tumour core.^13^ Pro‐angiogenic factors such as VEGF and Notch have historically been examined as potential therapies for cancers that are characterized by unregulated angiogenesis. For example, various strategies for inhibiting the VEGF pathway have been investigated. The most common therapy is bevacizumab, which inhibits the binding of VEGF onto its receptors, another is sunitinib which targets the VEGF receptor tyrosine kinase inhibitors (RTKIs).^9^, ^14^ Additionally, four clinical trials (NCT01122901, NCT01119599, NCT01269411 and NCT01189240) were conducted using RO4929097, a Notch signalling pathway inhibitor, against GBMs both as a single agent and in combination with TMZ or bevacizumab.^15^ However, from these trials, only one phase 1 trial was completed, while the other three were terminated due to the termination of drug supply from the manufacturer.

ELTD1 (epidermal growth factor, latrophilin and seven transmembrane receptor containing protein 1 on chromosome 1, ADGRL4) has previously been shown to be involved in brain angiogenesis and was shown to be regulated by the VEGF and DLL4/Notch signalling pathways.^16^ ELTD1 has higher expression in human high‐grade gliomas when compared to low‐grade gliomas.^17^ Moreover, targeting ELTD1 with an antibody was found to be effective in a G55 human GBM xenograft mouse model as demonstrated by decreasing tumour volumes, normalizing tumour vasculature and increasing survival.^18^, ^19^ Further optimization of the antibody therapy showed higher binding specificity against the tumour.^18^, ^19^

OKN‐007 (OKN), which was recently found to target the transforming growth factor β1 (TGF β1) pathway, is also a small molecule that is effective in crossing the blood‐brain barrier.^20^ From previous pre‐clinical studies in U87 and G55 GBM xenografts, and C6, GL261 and F98 high‐grade glioma animal models, it was established that OKN is an effective therapy against GBM/high‐grade gliomas by inhibiting cell proliferation and tumour necrosis, increasing apoptosis and increasing survival.^20^, ^21^, ^22^, ^23^, ^24^ Recently, it was found that when OKN is combined with TMZ, it increases TMZ sensitivity, thus increasing a significant effect on TMZ‐resistant GBM cells.^20^ OKN is also known to target tumour‐associated angiogenesis by targeting and decreasing both VEGFR2a and HIF‐1α protein expression.^21^, ^25^ Currently, OKN is in two GBM clinical trials, (1) phase II open‐label OKN combined with TMZ in patients with recurrent GBM and (2) early phase I OKN +TMZ concurrent treatment on patients with GBMs undergoing radiotherapy.

Despite the multimodal therapeutic approach to GBMs, the 5‐year relative survival rates are about 5%.^2^ Previous research has demonstrated that single‐agent anti‐ELTD1 and OKN‐007 therapies are effective in GBM pre‐clinical models. Therefore, in this study, we aimed to compare the two treatments against each other as well as against bevacizumab, an established GBM treatment in clinics.

## 2 MATERIALS AND METHODS

### 2.1 Generation of anti‐ELDT1 antibodies

Human ELTD1 (Glu20‐Leu406) and mouse ELTD1 (Glu20‐Leu455) genes were used to create the extracellular domains of human and mouse ELTD1 expression vectors as previously reported.^19^ Briefly, the expression vectors were transfected into HEK293F cells (Invitrogen, Carlsbad; CA, USA) and the C_kappa_ fusion proteins were purified from the supernatant using KappaSelect resin (GE Healthcare).

Chickens (white leghorn) were inoculated with a human ELTD1 C_kappa_ fusion protein, where the RNA was obtained from bone marrow, spleen and bursa of the chickens as previously described.^18^ Briefly, phage‐display library was created from the clones and the positive clones that showed cross‐reactivity with human and mouse ELTD1 were selected and enriched determined by Sanger sequencing.

Human or mouse ELTD1 C_κ_ fusion proteins were pipetted onto 96‐well microtiter plates (Corning Inc., Corning, NY, USA) in a coating buffer as previously described.^19^ 0.1 M NaHCO_3_ buffer (pH 8.6) was used to coat the ELTD1 C_κ_ fusion proteins, and then subsequently blocked with 3% (w/v) BSA in phosphate‐buffered saline (PBS). Microtitre plate wells were subsequently incubated and washed as previously reported.^19^

### 2.2 Glioma model and treatment

Animal studies were conducted with the approval (protocol 17–48) of the Oklahoma Medical Research Foundation Institutional Animal Care Use Committee policies, which follow NIH guidelines. Two‐month‐old male Athymic Nude‐Foxn1nu mice (Harlan Inc., Indianapolis, IN) were implanted with human G55 cells as previously described.^26^, ^27^ The animals were divided into 5 treatment arms: UT, mmAb anti‐ELTD1, OKN‐007, combined anti‐ELTD1 and OKN, and bevacizumab (Genentech). Upon tumour detection (6–7 mm^3^), the animals were left untreated or were administered with 2 mg/kg of the antibody treatments every 3–4 days (treated M/Th, T/F, W/Sat). OKN‐007 (2, 4‐disulfophenyl‐N‐tert‐butyl nitrone) was administered to the mice in their drinking water at a concentration of 125 mg/kg/day. The amount of OKN‐007 consumed by each mouse was determined by weighing water bottles each day. No significant deviation was observed in the volume of liquid uptake of compound in these mice. The average intake of OKN‐007 was approximately 140–150 mg/kg/day. All mice were euthanized when tumours reached ≥150 mm^3^.

### 2.3 *In vivo* magnetic resonance (MR) techniques

#### 2.3.1 Morphological imaging

All mice were subjected to MR imaging while under anaesthesia and restrained in a cradle which was inserted into a 30‐cm horizontal bore Brucker Biospin magnet (7T, Bruker BioSpin GmbH; Karlsruhe, Germany). The animals were first imaged 10 days post‐tumour implantation and then every 3–4 days depending on treatment administration with a BA6 gradient set and mouse coil as previously described.^26^

#### 2.3.2 Perfusion imaging

The perfusion imaging method, arterial spin labelling, was used as previously described.^28^ For perfusion quantification, five region of interests (ROIs) were outlined in the tumour as well as in the contralateral side of the brain as a control. Blood perfusion rates (BPRs) values were determined by subtracting late and early tumour BPRs. This difference was then normalized to BPRs in the contralateral brain region of corresponding animals.

### 2.4 Immunohistochemistry and standard staining

Five micron formalin‐fixed, paraffin‐embedded sections were mounted on HistoBond Plus slides (Statlab Medical Products, Lewisville, TX). Sections were rehydrated and washed in tris‐buffered saline (TBS). Rabbit antibodies were used for CD34 (cat# ab81289, 1:200, Abcam, Cambridge, MA), c‐Met (cat# sc‐10, 4 µg/ml, Santa Cruz Biotechnology, Santa Cruz, CA), Caspase‐3 (cat# sc‐7148, 4 µg/ml, Santa Cruz Biotechnology, Santa Cruz, CA), Cleaved Caspase‐3 (cat# 9661,1:400, Cell Signaling, Danvers, MA), Ki‐67 (cat# NB600‐1209, 2 µg/ml, Novus Biologicals, Littleton, CO), CD44v6 (cat# orb13319, 1.7 µg/ml, biorbyt Ltd, San Francisco CA), TRPM8 (cat# ab3243, 1 μg/ml, abcam, Cambridge, MA), BMP2 (cat# ab14933, 4 µg/ml, Cambridge, MA), Notch 1 (cat# ab52627, 11 µg/ml, abcam, Cambridge, MA), VEGF Receptor 2 (cat# ab2349, 1:100, abcam, Cambridge, MA), L1CAM (cat# ab270455, 1 µg/ml, abcam, Cambridge, MA). A mouse antibody for anti‐Mitochondria (cat# MAB1273, 1:500, Millipore, Temecula, CA) was used to stain mitochondria for human cells. For Immunohistochemistry, slides were processed using Anti‐Rabbit IgG ImmPRESS^®^ Excel Amplified Polymer kit Peroxidase (cat# MP7601, Vector Labs, Burlingame, CA) or Anti‐Mouse IgG ImmPRESS^®^ Excel Amplified Polymer kit Peroxidase, (cat# MP‐7602, Vector Labs Inc., Burlingame, CA). Antigen retrieval (pH 6 Citrate Antigen Unmasking Solution (cat# H‐3300, Vector Labs Inc., Burlingame CA) or a pH 9 tris‐based wash (cat# H3301, Vectors Labs, Burlingame, CA) was accomplished via 20 min in a steamer followed by 30 min cooling at room temperature. Sections were then treated with a peroxidase blocking reagent (Bloxall, cat# SP‐6000, Vector Laboratories, Inc, Burlingame, CA) to inhibit endogenous peroxidase activity, followed by 2.5% normal horse serum blocking reagent to inhibit nonspecific binding. Appropriate washes were then made in TBS. Antibodies were applied to each section, and following incubation overnight at 4°C in a humidified chamber, sections were washed in TBS. All reagents were applied according to the manufacturer's directions. Slides were incubated with NovaRed^®^ (Vector Laboratories, Inc., Burlingame, CA) chromogen for visualization. Counterstaining was carried out with Hematoxylin QS Nuclear Counterstain (Vector Laboratories; Burlingame, CA). Appropriate positive and negative tissue controls were used.

To characterize IHC positivity levels, five regions of interest (ROIs), captured digitally (20×) using a Leica‐Aperio CS whole slide scanner, were identified in each case. The vessel count algorithm from Leica‐Aperio ImageScope was used to count the vessel to determine the MVD. Only areas containing tumour tissue were analysed, excluding areas with necrosis and/or significant artefacts. The number of positive pixels was divided by the total number of pixels (negative and positive) in the analysed area. ROIs were analysed using Leica‐Aperio ImageScope and Leica‐Aperio Tool Box (pixel count algorithm) (Leica Biosystems, Buffalo Grove, IL).

### 2.5 Statistical analysis

Survival curves were analysed using Kaplan‐Meier curves. Tumour volumes, perfusion changes and immunohistochemistry protein levels were analysed and compared by one‐ or two‐way ANOVA with multiple comparisons (Tukey's or Sidak's, respectively). Data were represented as mean ± SD, and *p*‐values of either *<0.05, **<0.01, ***<0.001, ****<0.0001 were considered statistically significant.

## 3 RESULTS

**FIGURE 1:**
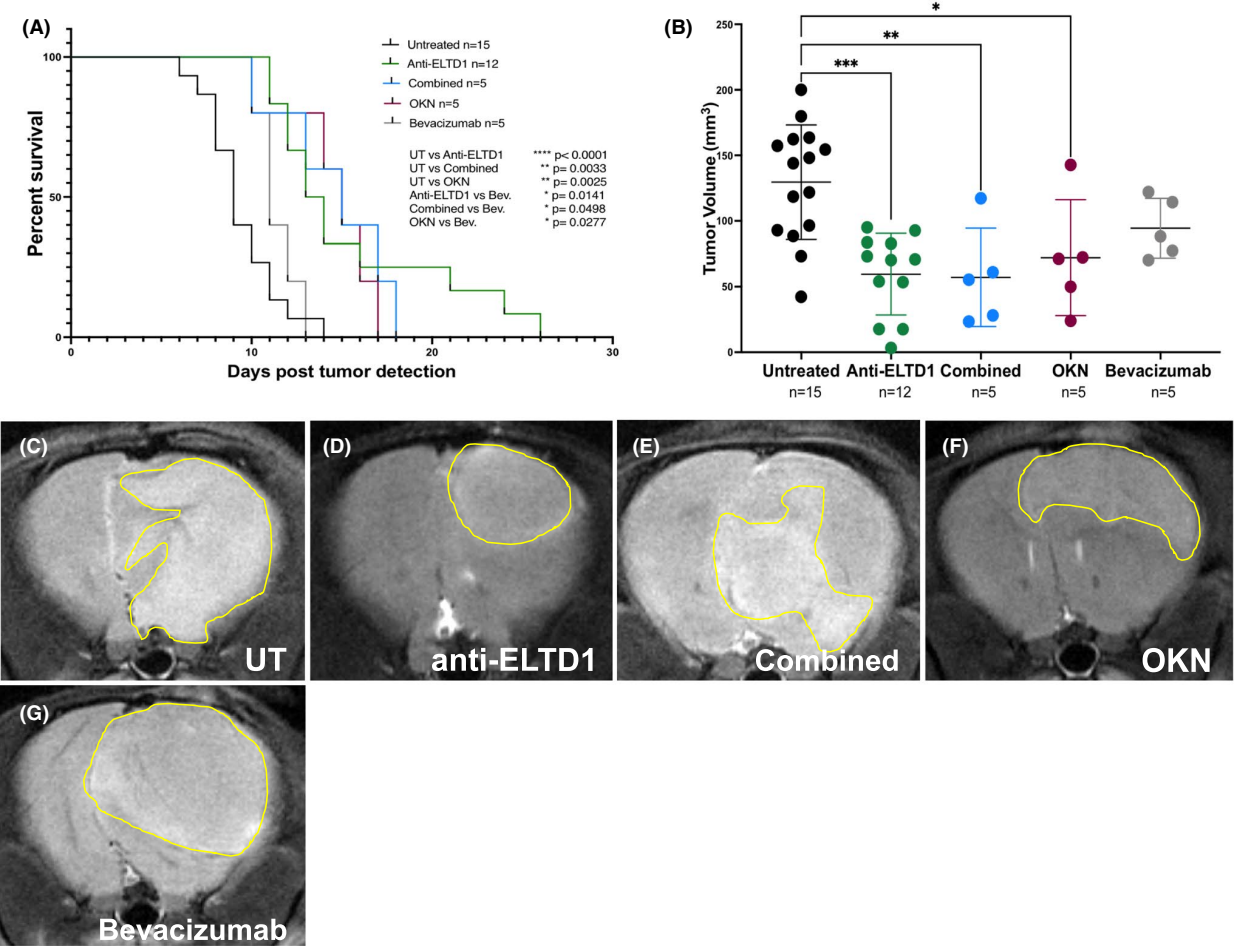
Anti‐ELTD1 and OKN treatments have similar effects on animal survival and TV. (A) Per cent survival curve for all treatment groups; untreated, anti‐ELTD1, OKN, combined anti‐ELTD1 and OKN, and bevacizumab treatments. Anti‐ELTD1 (****p < 0.0001), OKN (**p = 0.0033) and combined (**p = 0.0025) groups significantly increased the overall survival post‐tumour detection. (B) Tumour volumes of each treatment groups 9 days post‐tumour detection. (UT vs Anti‐ELTD1 ***p = 0.0003, UT vs OKN *p = 0.0416, UT vs combined **p = 0.0057) Representative morphological MR images for untreated (C), anti‐ELTD1 (D), OKN (E), combined (F), bevacizumab (G) and 9 days post‐tumour detection. Tumour boundaries are depicted with yellow lines

In this study, we aimed to compare OKN‐007 therapy and monovalent monoclonal antibody treatments against ELTD1 against the clinically used agent, bevacizumab. Tumour growth was monitored via MRI every 3–4 days, and the animals were either left untreated or treated via tail vein upon tumour detection with either mmAb anti‐ELTD1 Ab or bevacizumab (2 mg/kg), or given OKN via the drinking water. Once the tumours reached 150 mm^3^ (via MRI) mice were sacrificed, and their brains were harvested for histology. The untreated animal and ELTD1 treatment group data were obtained from cumulative studies. As shown in Figure 1A, untreated animals had an average survival of 9 days post‐tumour detection. Bevacizumab treatment did not significantly affect percentage of survival and generated an average animal survival of 11 days post‐tumour detection. On the other hand, single‐agent mmAb anti‐ELTD1 and OKN treatments were successful in significantly increasing the overall per cent survival compared to untreated (****p < 0.0001 and **0.0025, respectively) and bevacizumab (*p = 0.0141 and *p = 0.0277, respectively)‐treated animals. Anti‐ELTD1 treatment had an average survival of 16 days post‐tumour detection meanwhile OKN treatment increased the average survival to 14 days. Combining anti‐ELTD1 and OKN treatments significantly increased survival compared to both untreated and bevacizumab‐treated animals (**p = 0.0033 and *p = 0.0498, respectively). However, there was no additive effect of combining anti‐ELTD1 and OKN therapies and therefore no significant difference between the single‐agent effect on animal survival compared to the combined therapy.

Animal tumour volumes were assessed based on the average survival of untreated animals. As shown in Figure 1B, untreated animals had an average tumour volume of 130 mm^3^ at day 9 post‐tumour detection. Bevacizumab‐treated animals did not significantly decrease the tumour volumes and had an average tumour volume of 94 mm^3^. Similar to overall survival, anti‐ELTD1, OKN and combined groups significantly reduced (***p = 0.0003, *p = 0.0416, **p = 0.0057, respectively) tumour volumes when compared to untreated animals. Average animal tumour volumes 9 days post‐tumour detection can be seen in the representative MR images shown in Figure 1C–G.

**FIGURE 2:**
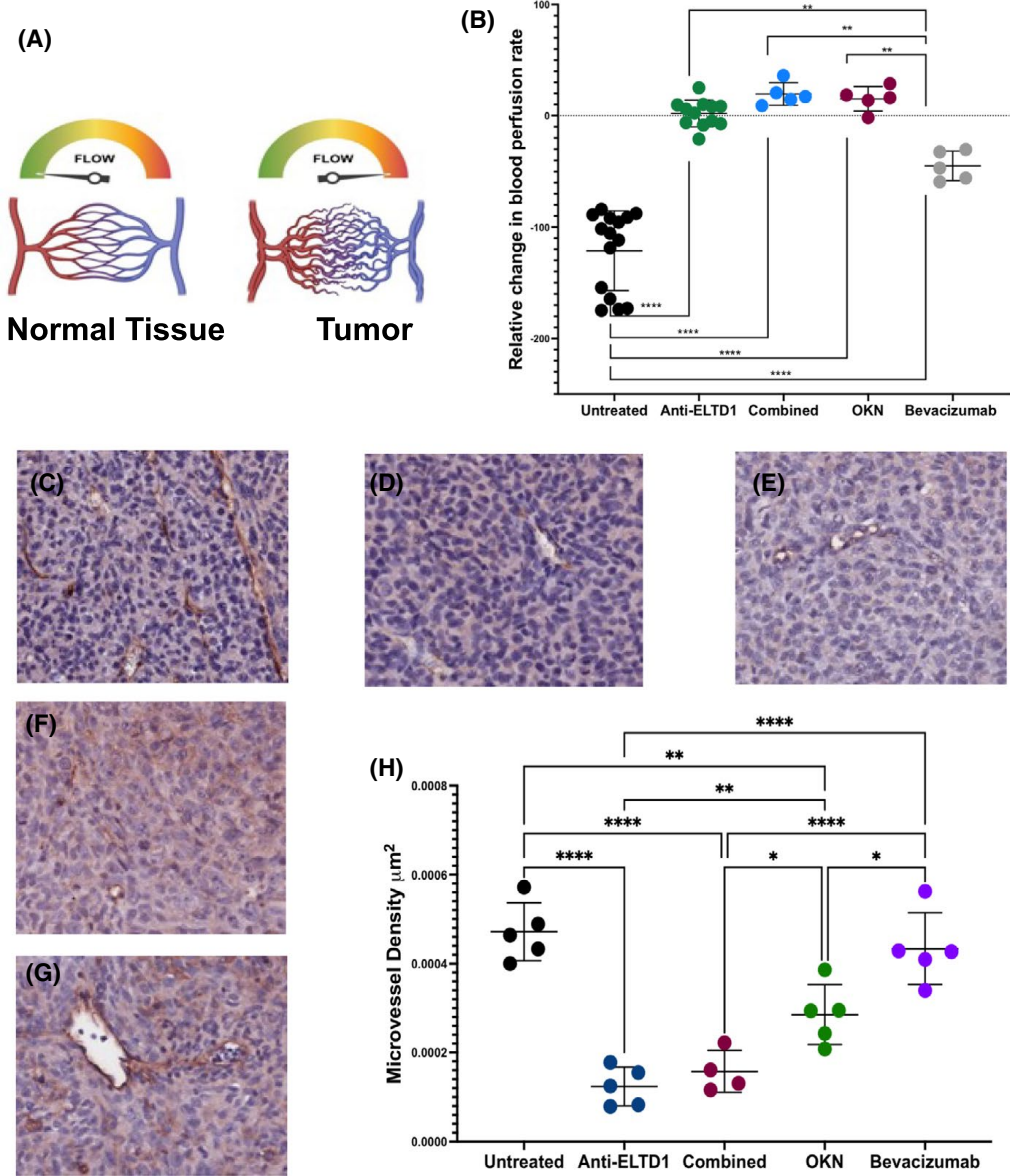
Anti‐ELTD1 and OKN treatments normalize the tumour‐associated vasculature within the tumour. (A) Perfusion schematic. The vasculature has a set perfusion rate in normal tissue due to the organized vasculature. The perfusion value decreases as the vasculature within the tumour becomes chaotic. (B) Quantitative analysis of tumour relative blood perfusion rates differences. The perfusion levels were significantly increased with both anti‐ELTD1 treatments. Anti‐ELTD1, OKN and combined therapies normalized the perfusion levels. (anti‐ELTD1: UT ****p < 0.0001, bevacizumab **p = 0.0061; OKN: UT ****p < 0.0001, bevacizumab **p = 0.0027; combined: UT ****p < 0.0001, bevacizumab **p = 0.0012). Representative IHC images (20×) for CD34 from untreated (C), anti‐ELTD1 (D), combined (E), OKN (F) and bevacizumab (G)‐treated animals. Dark red/brown staining in the slides represent vessels in the tumour region. (H) MVD analysis for all of the treatment groups

To assess microvasculature alterations within the tumour region, we used MRI perfusion to measure the relative change in BPRs. As shown in Figure 2A, normal tissue has an organized vasculature and therefore has a set BPR. However, due to increased angiogenesis, the vasculature becomes chaotic and decreases the perfusion rates within the tissue.^29^ The untreated animals had a drastic decrease in BPR, shown in the quantitative graph in Figure 2B, which depicts an increase of angiogenesis within the tumour region. Anti‐ELTD1 and OKN treatments both normalized tumour vascular perfusion rates compared to untreated and bevacizumab (anti‐ELTD1: UT ****p < 0.0001, bevacizumab **p = 0.0061, OKN: UT ****p < 0.0001, bevacizumab **p = 0.0027). There was significant normalization of the perfusion rates between the combined therapy against both untreated and bevacizumab‐treated animals; however, there was no additive effect with the combined therapy (UT ****p < 0.0001, bevacizumab **p = 0.0012). Therefore, we can conclude that anti‐ELTD1 and OKN treatments are more effective in normalizing the microvasculature alterations associated with GBMs.

To further examine the effect of each treatment on tumour‐associated vasculature, we analysed microvascular density (MVD) of each tumour sample staining for CD34. CD34 is a well known endothelial cell marker for the quantification of angiogenesis.^30^, ^31^ Representative CD34 IHC images are shown in Figure 2C–G. MVD analysis shown in Figure 2H demonstrated that combined treatment significantly decreased MVD within tumour regions compared to untreated, bevacizumab and OKN‐treated samples (UT and bevacizumab ****p < 0.0001, OKN *p = 0.01). This effect, however, does not seem to be an additive effect from anti‐ELTD1 and OKN treatments. Instead, this effect may be mainly attributed to the properties of the anti‐ETLD1 as anti‐ELTD1 treatment also significantly decreased MVD levels compared to untreated, bevacizumab and OKN treatments (UT and bevacizumab ****p < 0.0001, OKN **p = 0.0055). Although OKN therapy was less effective in decreasing MVD compared to anti‐ELTD1, it was still more effective when compared to untreated and bevacizumab therapy (UT ****p < 0.0001, bevacizumab *p = 0.011).

**FIGURE 3:**
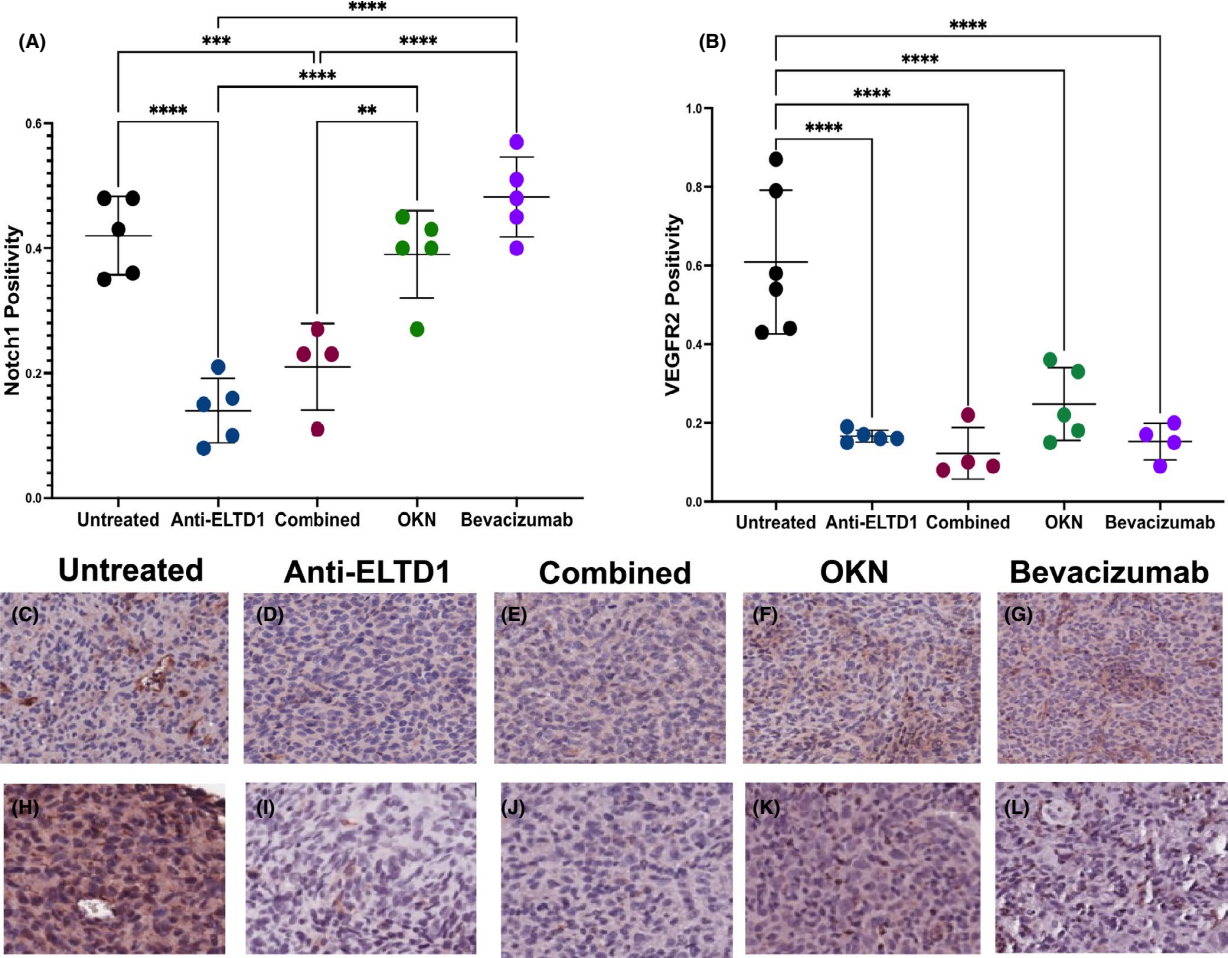
Anti‐ELTD1 therapy significantly decreased Notch1 and VEGFR2 levels, while OKN treatment only targeted VEGFR2 positivity levels. Quantitative analysis of Notch1 (A) positivity within the tumour region. (anti‐ELTD1: UT, bevacizumab and OKN ****p < 0.0001; combined: UT ***p = 0.0008, bevacizumab ****p < 0.0001, OKN **p = 0.0038) and VEGFR2 (B) staining of the samples. (****p < 0.0001). Representative IHC images (20×) for Notch1 from untreated (C), anti‐ELTD1 (D), combined (E), OKN (F) and bevacizumab (G)‐treated animals. Representative images (20×) of IHC stained tumours with VEGFR2 of untreated (H), anti‐ELTD1 (I), combined (J), OKN (K) and bevacizumab (L)

The next question then becomes, what is happening with the pro‐angiogenic factors, Notch1 and VEGFR2, within the tumour region. Deregulation of the Notch signalling is crucial for tumour development and progression; therefore, we investigated what effects each treatment had on Notch1 positivity levels. The untreated G55 pre‐clinical mouse model demonstrates an upregulation of Notch1 staining having an average of 41% Notch1 positivity staining within the tumour region. Bevacizumab treatment did not affect Notch1 staining and also had an average of 48% Notch1 positivity staining, as shown in Figure 3A. As shown in the quantitative graph and representative images in Figure 3A, anti‐ELTD1 and combined treatments were successful in significantly decreasing Notch1 positivity staining compared to untreated, bevacizumab and OKN‐treated animals (anti‐ELTD1: UT, bevacizumab and OKN ****p < 0.0001; combined: UT ***p = 0.0008, bevacizumab ****p < 0.0001, OKN **p = 0.0038). There was no significant difference in Notch1 positivity staining when comparing OKN to untreated and bevacizumab‐treated animals (see representative images in Figures C‐G). The next angiogenic marker in question was VEGFR2. Similar to Notch1, VEGFR2 positivity levels were upregulated, with an average of 61%, in the untreated animals as shown in Figure 3B. As expected, bevacizumab significantly decreased VEGFR2 positivity levels within the tumour region compared to untreated (UT ****p < 0.0001). Additionally, anti‐ELTD1, OKN and combined therapies also significantly decreased VEGFR2 positivity compared to untreated controls (anti‐ELTD1 and combined: UT ****p < 0.0001; OKN: UT ***p = 0.0002). There was no significant difference between the four treatment groups as shown through the representative images (Figure 3H–L).

**FIGURE 4:**
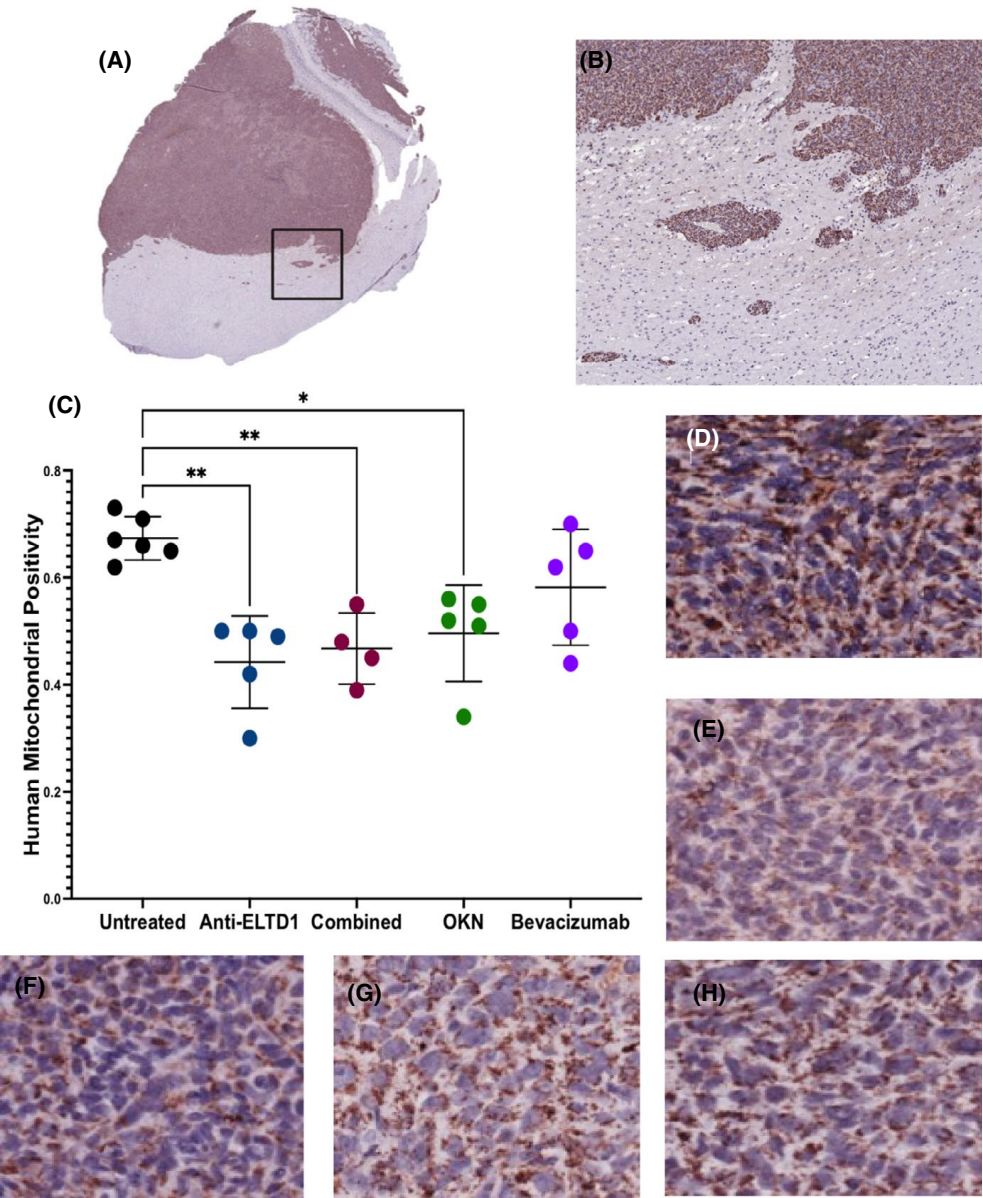
Anti‐ELTD1 and OKN therapies specifically target human tumour cells. A. Untreated tumour tissue showing human mitochondrial antibody staining (1×). The black box corresponds to (B) and shows that there is no mitochondrial staining in the normal brain tissue (5×). Quantitative analysis of human mitochondrial antibody (C) staining of each group. IHC representative images (20×) of untreated (D), anti‐ELTD1 (E), combined (F), OKN (G), bevacizumab (H). (anti‐ELTD1 vs UT **p = 0.0011; combined vs UT **p = 0.0062; OKN vs UT *p = 0.0128)

We wanted to determine if the effects we were seeing were due to the direct targeting of the human tumour cells (G55 GBM cells). Therefore, we stained the tumour tissue against the human mitochondrial antibody. Figure 4A,B is a representative untreated IHC slice in 1× and 5×, respectively, showing that there is no human mitochondrial antibody staining in the healthy contralateral tissue. The black box and Figure 4B shows the distinct tumour boundaries between the tumour/infiltrating cells and the healthy mouse tissues. Quantification of the untreated animals demonstrates a robust overexpression of human mitochondrial positivity staining within the G55 tumour region (Figure 4C). Anti‐ELTD1, OKN and combined therapies, but not bevacizumab, were successful in decreasing the human mitochondrial positivity staining within the tumour region compared to untreated (Figure 4D–H) (anti‐ELTD1 vs UT **p = 0.0011; combined vs UT **p = 0.0062; OKN vs UT *p = 0.0128).

**FIGURE 5:**
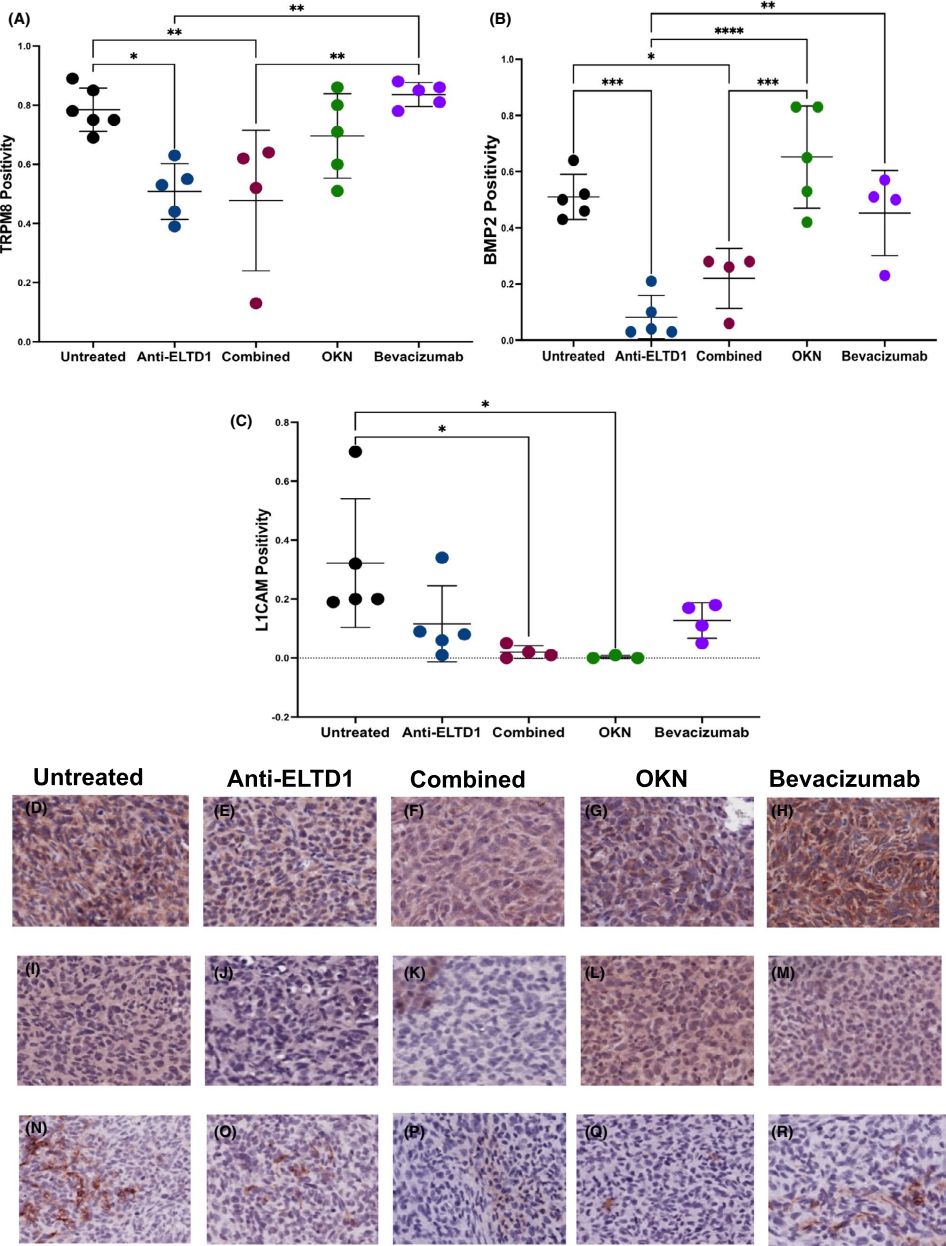
Anti‐ELTD1 and OKN therapies affect and decrease different migration markers. Quantitative analysis of TRPM8 (A), BMP2 (B) and L1CAM (C) staining of each group. TRPM8 IHC representative images (20×) of untreated (D), anti‐ELTD1 (E), combined (F), OKN (G), bevacizumab (H). Representative images (20×) of BMP2 stained tissue samples of untreated (I), anti‐ELTD1 (J), combined (K), OKN (L), bevacizumab (M). L1CAM IHC representative images (20×) of untreated (N), anti‐ELTD1 (O), combined (P), OKN (Q), bevacizumab (R). TRPM8: anti‐ELTD1: UT *p = 0.0132, bevacizumab **p = 0.0045; combined: UT **p = 0.0095, bevacizumab **p = 0.0034. BMP2: anti‐ELTD1: UT ***p = 0.0004, bevacizumab **p = 0.0029, OKN ****p < 0.0001). Combined: UT *p = 0.0217, OKN ***p = 0.0006. L1CAM: OKN vs UT *p = 0.0279; combined vs UT *p = 0.0226

Bevacizumab studies have demonstrated that therapies that target angiogenesis in GBMs cause an increase of invasiveness by promoting a migratory phenotype to ensure sufficient delivery of oxygen.^8^, ^14^ This, therefore, led us to investigate if our other treatments favoured a pro‐migratory phenotype. The transient receptor potential melastatin family member 8 (TRPM8), bone morphogenetic protein 2 (BMP2) and L1CAM are upregulated in GBMs compared to normal brain tissue and are associated with glioma cell proliferation, migration and invasion.^32^, ^33^, ^34^, ^35^ To assess tumour cell migration, we first stained the tumour tissue against TRPM8. As shown in Figure 5A, untreated animals had high TRPM8 signalling positivity with an average of 78%. Both bevacizumab and OKN treatments did not have an effect on TRPM8 and instead bevacizumab had an increased average positivity of TRPM8 to 84% while OKN treatment (70%) had a slight decrease from untreated. On the other hand, anti‐ELTD1 and combined therapies were all successful in significantly decreasing TRPM8 positivity staining within the tumour region (anti‐ELTD1: UT *p = 0.0132, bevacizumab **p = 0.0045; combined: UT **p = 0.0095, bevacizumab **p = 0.0034) (Figure 5D–H).

The next migratory marker we investigated was BMP2. Similar to TRPM8, the untreated animals showed an upregulation of BMP2 positivity staining within tumour regions. Again, there was no significant difference between untreated BMP2 levels and bevacizumab or OKN (Figure 5B,I–M). However, we did see a significant decrease between anti‐ELTD1 and three of the other groups (untreated, bevacizumab and OKN) (UT ***p = 0.0004, bevacizumab **p = 0.0029, OKN ****p < 0.0001). Combined anti‐ELTD1 and OKN therapy were also successful in decreasing BMP2 levels compared to untreated and OKN treated (UT *p = 0.0217, OKN ***p = 0.0006).

Unlike the previous two migratory markers, we did not see a robust overexpression of L1CAM in the tumour tissue of untreated animals (32% positivity). Although there was a downward trend of L1CAM positivity with both anti‐ELTD1 (11% positivity) and bevacizumab (12% positivity) treatments, there was no significant difference between the two treatments and untreated. Furthermore, both OKN (*p = 0.0279) and combined (*p = 0.0226) therapies were successful in significantly decreasing L1CAM positivity compared to untreated. These two treatments were also successful in completely eradicating L1CAM positivity within the tumour region (Figure 5C,N–R).

**FIGURE 6:**
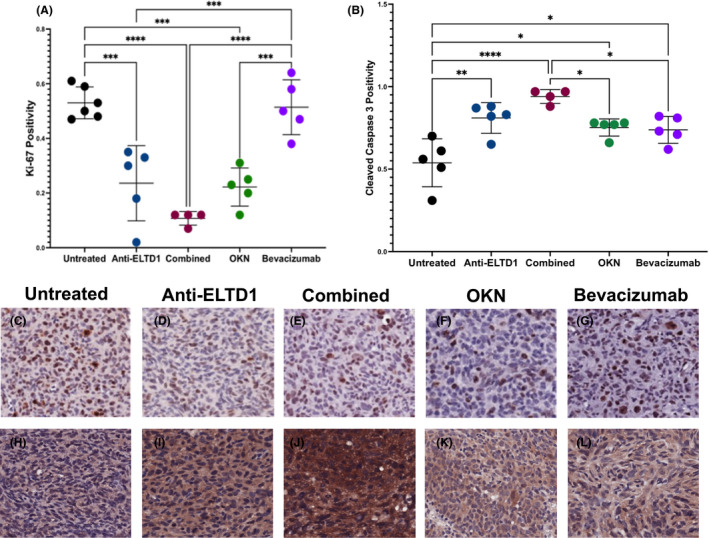
Anti‐ELTD1 and OKN therapies decrease cellular proliferation and induce apoptosis. Quantitative analysis of Ki‐67 (A) and cleaved caspase 3 (B) staining of each group. Ki‐67 IHC representative images (20×) of untreated (C), anti‐ELTD1 (D), combined (E), OKN (F), bevacizumab (G). Untreated vs anti‐ELTD1 ***p = 0.0002, OKN ***p = 0.0001, combined ****p < 0.0001 and bevacizumab vs. anti‐ELTD1 ***p = 0.0006, OKN ***p = 0.0003, combined ****p < 0.0001. Cleaved caspase 3 IHC representative images (20×) of untreated (H), anti‐ELTD1 (I), combined (J), OKN (K), bevacizumab (L). Untreated vs. Anti‐ELTD1 (**p = 0.0014), combined (****p < 0.0001), OKN (*p = 0.0126) and bevacizumab (*p = 0.0211), combined therapy compared to bevacizumab (*p = 0.0297) and OKN (*p = 0.0473)

We then sought to determine if the various treatments had an effect on cellular proliferation within the tumour region. Ki‐67 is an established cell proliferation marker that has also been strongly correlated with GBM tumour growth and metastasis.^36^ As shown in Figure 6A, both untreated (53%) and bevacizumab (51%)‐treated animals had high per cent positivity for Ki‐67 staining within the tumour region. Anti‐ELTD1, OKN and combined therapies were all successful in decreasing Ki‐67 positivity compared to both untreated (anti‐ELTD1 ***p = 0.0002, OKN ***p = 0.0001, combined ****p < 0.0001) and bevacizumab (anti‐ELTD1 ***p = 0.0006, OKN ***p = 0.0003, combined ****p < 0.0001) as shown through the representative images in Figure 6C–G. While there was no significant difference between anti‐ELTD1, OKN or combined therapies, combined therapy (11%) seems to have a more significant downward trend when looking at the average Ki‐67 positivity within the tumour region when compared against anti‐ELTD1 (24%) and OKN (22%) treated.

Aside from tumour angiogenesis, migration and cell proliferation, we wanted to investigate if our therapies had any effect on apoptosis. We stained the tumour tissue against cleaved caspase 3, the activated form of caspase 3, which is used to evaluate the presence of apoptosis within a given tissue. As shown in Figure 6B, the untreated animals had an average cleaved caspase 3 activity of 54% within the tumour region. All of the treatment groups, Anti‐ELTD1 (**p = 0.0014), combined (****p < 0.0001), OKN (*p = 0.0126) and bevacizumab (*p = 0.0211), were successful in increasing the apoptotic activity within the tumours. Additionally, as seen through the representative images in Figure 6H–L, combined therapy had a more significant effect in inducing apoptotic activity compared to both bevacizumab (*p = 0.0297) and OKN (*p = 0.0473).

**FIGURE 7:**
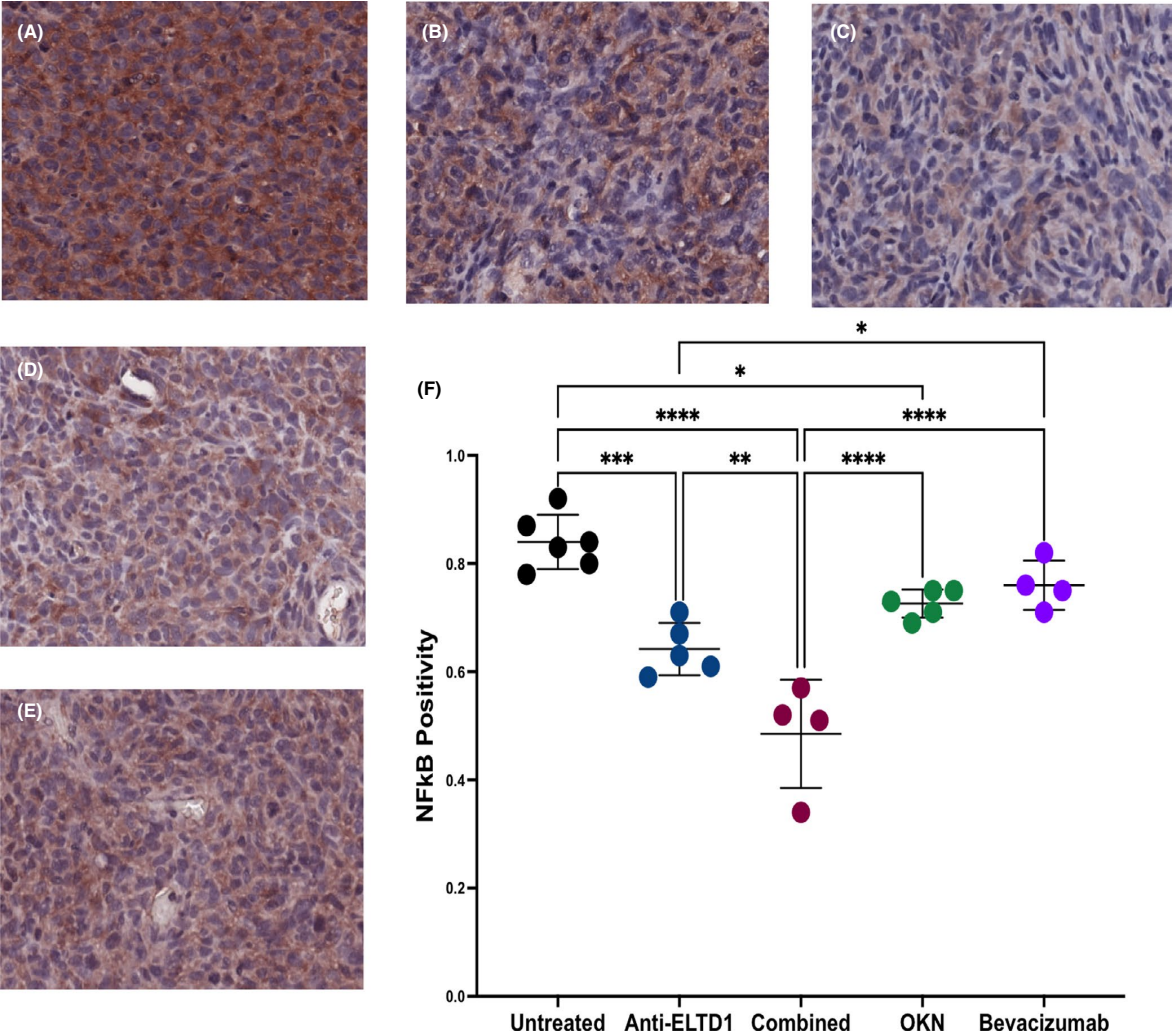
Combined anti‐ELTD1 and OKN therapies significantly decrease NF‐κB positivity expression levels. IHC representative images (20×) of untreated (A), anti‐ELTD1 (B), combined (C), OKN (D), bevacizumab (E). Quantitative analysis of NF‐κB (F) staining of each group. Anti‐ELTD1: UT ***p = 0.0001, bevacizumab *p = 0.0408; OKN: UT *p = 0.26). Combined: UT, OKN and bevacizumab ****p < 0.0001, anti‐ELTD1 **p = 0.0045

NF‐κB is important for various aspects of cancer biology including resistance to treatment, tumour growth and metastasis.^37^ G55 untreated and bevacizumab‐treated animals had robust NF‐κB positivity expression levels in the tumour region. However, as shown in Figure 7, both anti‐ELTD1 and OKN therapies were successful in decreasing NF‐κB positivity (anti‐ELTD1: UT ***p = 0.0001, bevacizumab *p = 0.0408; OKN: UT *p = 0.26). Furthermore, this is the first marker in which we begin to see an additive effect with the combined therapy (UT, OKN and bevacizumab ****p < 0.0001, anti‐ELTD1 **p = 0.0045). Representative images are shown in Figure 7A–E.

## 4 DISCUSSION

This study sought to compare anti‐ELTD1 and OKN therapies in an *in vivo* G55 xenograft mouse study. MRI is a powerful tool used by clinicians to monitor tumour progression and clinical effectiveness of prescribed treatment regimens.^38^ In this study, we used morphological and perfusion MRI to compare anti‐ELTD1 and OKN therapies. Both anti‐ETLD1 and OKN treatments were significantly better at increasing overall survival when compared to untreated, and there was no significant difference between the two groups. Furthermore, there was also no significant difference between the two groups with overall tumour volumes.

Typically, the tumour‐associated microvasculature alterations associated with tumour angiogenesis is measured by perfusion MRI, which measures a relative change in BPR, and is used to assess the efficacy of anti‐angiogenic therapies.^39^ In GBMs, the tumour‐associated vasculature has irregular and leaky vessels that cause the BPR to be lower than the surrounding normal brain.^40^ Once treated with anti‐angiogenic therapies, the BPRs within the tumour tend to increase in patients. An explanation to this observation is that there is a decrease of the leaky vessels created by the tumour and instead the remaining vessels mimic normal vessels which results in increased blood perfusion.^40^ In regard to tumour vascularization, our results showed a drastic decrease in perfusion rates in untreated animals, which is characteristic sign of increased angiogenesis. Although bevacizumab was capable of decreasing this drastic change in perfusion rate, it seems that bevacizumab therapy may not be as effective in normalizing microvascularity within the tumour regions compared to anti‐ELTD1 and OKN therapies. Anti‐ELTD1 therapy was more successful in decreasing microvessel density levels. Although there was no additive effect with the combined therapy in any of these aspects, MVD levels were significantly decreased compared to untreated, bevacizumab and OKN therapies. Again, our results demonstrate that bevacizumab therapy has no significant effect on the microvascular density levels within the tumour region.

In normal vasculature, ELTD1 expression is regulated by VEGF and Notch/DLL4 pathways.^16^ We previously examined the relationship between VEGF and a polyclonal anti‐ELTD1 antibody or OKN‐007 treatment. In those studies, we demonstrated that ELTD1 and VEGFR2 were co‐localized within blood vessels and glioma cells in G55 glioma tumours. The polyclonal anti‐ELTD1 treatment significantly reduced the expression of ELTD1 and VEGFR2, but had no significant effect on VEGF.^27^ In orthotopic rat F98 and human U87 xenograft glioma models, OKN‐007 was shown to decrease microvessel density levels as shown through CD31; however, OKN‐007 did not alter VEGF levels.^41^ In this study, we further demonstrated that in a GBM model, targeting ELTD1 results in decreased expression of both VEGFR2 and Notch1. This further demonstrates that anti‐ELTD1 treatment works to decrease several pro‐angiogenic factors (although not VEGF) within the tumour region. OKN‐007 treatment was also effective in decreasing VEGFR2 expression levels, however, had no effect on Notch1. This further demonstrates that anti‐ELTD1 treatment is directly associated with the Notch signalling pathway.

Previous studies have shown that anti‐angiogenic therapies, such as bevacizumab, cause GBMs to become progressively invasive and invade normal brain tissue leading to the formation of satellite tumours.^6^ In this study, we sought to determine whether various treatments affected the invasiveness of the tumours by examining the proliferative rate and the expression of various migratory markers. Bevacizumab treatment had no effect on various migration markers compared to untreated and had a high Ki‐67 positivity staining. This suggests that bevacizumab treatment is not effective in decreasing tumour invasiveness.

However, anti‐ELTD1 and OKN treatments both significantly decreased Ki‐67 positivity suggesting that the tumour is has decreased invasive properties. Additionally, we see that anti‐ELTD1 and OKN treatments successfully targeted and decreased migration in our G55 pre‐clinical model. Anti‐ELTD1 therapy decreased TRPM8 and BMP2, while OKN therapy was successful in decreasing L1CAM positivity levels. This suggests that while anti‐ELTD1 and OKN therapies both target migratory pathways, they may have effects on different migration cellular pathways. It was previously shown from microarray analysis that OKN affects tumorigenesis by targeting the TGF‐β1 pathway by downregulating key‐associated genes has.^20^ OKN was shown to downregulate key genes associated with tumorigenesis via microarray analysis and is thought to target the TGF‐β1 pathway.^20^ Recently, evidence has suggested that the TGF‐β and BMP2 pathways may play opposing roles in cancer.^42^ This may explain why OKN‐007 did not have a significant effect on BMP2 positivity expression.

Anti‐ELTD1 and OKN therapies were both successful in decreasing cellular proliferation and inducing apoptosis within the tumours. Although there was no significant difference within the cellular proliferation marker between the combined treatment compared to the single‐agent use of anti‐ELTD1 and OKN, there is a downward trend associated with the combined group. Additionally, we do see that the combined treatment is more effective in inducing apoptosis when compared to OKN alone. This therefore provides another advantage for combined therapy. Furthermore, both anti‐ELTD1 and OKN therapies were successful in decreasing NF‐κB positivity levels. The combined therapy showed an additive effect by decreasing NF‐κB positivity expression levels from 84% expression in untreated animals to 49%. This finding suggests that both anti‐ELTD1 and OKN treatments target the NF‐κB signalling pathway. NF‐κB is a well known inflammation marker, and both anti‐ELTD1 and OKN therapies may have anti‐inflammatory effects. OKN has been shown to be an anti‐inflammatory agent for age‐associated neuroinflammation in a lipopolysaccharide (LPS)‐induced encephalopathy rat model.^43^ Currently, there is little data regarding the relationship between ELTD1 and inflammation, but future studies are needed to investigate this relationship. Further studies are being conducted to determine if Notch1 may be the master regulator of anti‐ELTD1 in GBM. Previous RNA‐sequencing studies indicate that Notch1 may be the master regulator of ELTD1. For instance, our data have demonstrated that key genes targeted by anti‐ELTD1 antibody therapy, such as c‐MET, Ki‐67, BMP2 and VEGFR2 are all correlated with Notch1.^18^, ^19^ Anti‐ELTD1 therapy has targeted key genes such as c‐MET, Ki‐67, BMP2 and VEGFR2 which are correlated with Notch1. Therefore, we have reason to believe that Notch1 may be the master regulator of ELTD1.^18^, ^19^

Further studies will need to be done to confirm the role of Notch 1 in ELTD1 antibody therapy.

## 5 CONCLUSION

In preliminary assessments, we saw that anti‐ELTD1 and OKN treatments were effective as therapies in a human G55 pre‐clinical model. In this paper, we validated those claims by demonstrating that both treatments increased survival, decreased tumour volumes, normalized the tumour‐associated vasculature, decreased migratory markers and specifically targeted human tumour cells. Anti‐ELTD1 and OKN‐007 seem to be similar in most instances; however, combined therapy seems to be more effective than either regarding NF‐κB or better than OKN‐007 in inducing apoptosis (cleaved caspase 3).

## CONFLICT OF INTEREST

Dr. Towner holds patents for OKN‐007 and ELTD1 as a target for GBM. None of other authors have any conflict of interest.

## AUTHOR CONTRIBUTIONS

**Michelle Zalles:** Conceptualization (supporting); data curation (lead); formal analysis (lead); methodology (lead); visualization (lead); writing – original draft (lead). **Nataliya Smith:** Data curation (supporting); investigation (supporting); methodology (supporting); supervision (supporting); writing – review and editing (equal). **Debra Saunders:** Methodology (supporting); supervision (supporting); writing – review and editing (equal). **Megan Lerner:** Methodology (supporting); resources (supporting); writing – review and editing (equal). **Kar‐Ming Fung:** Funding acquisition (supporting); methodology (supporting); resources (supporting); writing – review and editing (equal). **James Battiste:** Validation (supporting); writing – review and editing (equal). **Rheal A. Towner:** Conceptualization (lead); funding acquisition (lead); investigation (lead); project administration (lead); resources (lead); supervision (lead); validation (lead); writing – review and editing (lead).

## Data Availability

The data that support the findings of this study are available from the corresponding author upon reasonable request.
